# Systemic dengue infection associated with a new dengue virus type 2 introduction in Brazil – a case report

**DOI:** 10.1186/s12879-021-05959-2

**Published:** 2021-04-01

**Authors:** Marielton dos Passos Cunha, Amaro Nunes Duarte-Neto, Shahab Zaki Pour, Ludhmila Abrahão Hajjar, Fernando Pereira Frassetto, Marisa Dolhnikoff, Paulo Hilario do Nascimento Saldiva, Paolo Marinho de Andrade Zanotto

**Affiliations:** 1 Laboratory of Molecular Evolution and Bioinformatics, Department of Microbiology, Biomedical Sciences Institute, University of São Paulo, São Paulo, Brazil; 2 Pathology Department, Medical School, University of São Paulo, São Paulo, Brazil; 3 Intensive Care Unit, Heart Institute (InCor), Medical School, University of São Paulo, São Paulo, Brazil

**Keywords:** Dengue virus, Dengue virus type 2, Autopsy, Fatal case, Case report

## Abstract

**Background:**

Dengue infection is caused by an arbovirus with a wide range of presentations, varying from asymptomatic disease to unspecific febrile illness and haemorrhagic syndrome with shock, which can evolve to death. In Brazil, the virus circulates since the 1980s with many introductions of new serotypes, genotypes, and lineages since then. Here we report a fatal case of dengue associated with a Dengue virus (DENV) lineage not detected in the country until now.

**Case presentation:**

The patient, a 58-year-old man arrived at the hospital complaining of fever and severe abdominal pain due to intense gallbladder edema, mimicking acute abdomen. After 48 h of hospital admission, he evolved to refractory shock and death. DENV RNA was detected in all tissues collected (heart, lung, brain, kidney, spleen, pancreas, liver, and testis). Viral sequencing has shown that the virus belongs to serotype 2, American/Asian genotype, in a new clade, which has never been identified in Brazil before. The virus was phylogenetically related to isolates from central America [Puerto Rico (2005–2007), Martinique (2005), and Guadeloupe (2006)], most likely arriving in Brazil from Puerto Rico.

**Conclusion:**

In summary, this was the first fatal documented case with systemic dengue infection associated with the new introduction of Dengue type 2 virus in Brazil during the 2019 outbreak.

**Supplementary Information:**

The online version contains supplementary material available at 10.1186/s12879-021-05959-2.

## Background

Dengue virus (DENV) is the world’s most important arbovirus in terms of morbidity, mortality, and economic impact for humans [1]. DENV are arthropod-borne viruses associated with *Aedes* vectors [2], belong to the genus *Flavivirus* (family *Flaviviridae*) [3, 4], and are classified in four phylogenetically and antigenically distinct serotypes (DENV-1-4) that cause outbreaks in humans and are also established sylvatic cycle infecting non-human primates. In the last decades, a strong increase in prevalence occurred in tropical and subtropical areas worldwide [1, 5]. Likewise, in Brazil, a growing number of cases are notified every year, and the country is experiencing hyperendemic DENV circulation [6, 7]. Since DENV reintroduction in the early 1980s, continuous reintroductions were reported for the four viral serotypes [8–10], allowing the persistence of intense viral circulation in Brazil. About 60% of all dengue cases reported worldwide are observed in Brazil [6].

DENV-2 has six genetically known genotypes: (*i*) American; (*ii*) American/Asian; (*iii*) Asian 1; (*iv*) Asian 2; (*v*) Cosmopolitan; and (*vi*) Sylvatic [11, 12]. Recently, a group of basal DENV-2 highly divergent sequences was reported, which was considered by some virologists and geneticists a new genotype within the serotype [13, 14]. In Brazil, the recently reported cases are of the American [7] and American/Asian genotypes [15, 16], the latter being known for its more severe clinical manifestations at the acute phase of infection [17]. The American/Asian genotype was introduced in the Americas coming from Asia during the early 1980s, spreading widely into the continent [16]. After 1990 the virus was reintroduced in Brazil, by different strains from the Caribbean region [15, 16, 18]. Brazil has experienced a sizeable increase in dengue cases associated with DENV-2 in 2019. Until June 2019 (epidemiological week 26), the number of notifications continued to rise, reaching 1,281,759 cases, and 443 deaths [19]. Here, we report clinical, pathological, virological, and genomic findings associated with a fatal case caused by DENV-2.

## Case presentation

### Clinical case description

At the end of April 2019, a 58-year-old man arrived at the emergency department complaining of 3 days of fever (38 °C), headache, myalgia, and arthralgia, with severe abdominal pain on the right upper abdomen, nausea, vomiting, and dehydration during the last 24 h. He had a past medical history of pulmonary tuberculosis treated in 2014 and B2 thymoma treated with chemotherapy and pleuropneumonectomy in 2017. During the first hours of observation, the patient developed hypotension, requiring vasoactive drugs, and was transferred to the intensive care unit. The initial laboratory tests showed Hb = 15.0 g/dL [Reference Range (RR) = 13–18 g/dL], HT = 43.3% (RR = 40–52%); peripheral blood total leukocytes = 5800/mm^3^ (RR = 4000-11,000/mm^3^); neutrophils count = 5200/mm^3^ (RR = 1600-7000/mm^3^); lymphocytes = 300/mm^3^ (RR = 900–3400/mm^3^); platelets = 9000/mm^3^ (RR = 150,000-450,000/mm^3^); urea 122 mg/dL (RR = 10–50 mg/dL); direct bilirubin 1,58 mg/dL (RR < 0,3 mg/dL); alanine aminotransferase = 91 U/L (RR < 41 U/L); aspartate aminotransferase = 270 U/L (RR < 37 U/L); creatine phosphokinase = 36 U/L (RR = 39–308 U/L); arterial lactate = 59 mg/dL (RR = 4.5–14.4 mg/dL). Abdominal ultrasound showed thickened gallbladder wall (measuring 0.9 cm), pericholecystic fluid collection, liquid in the parieto-colic gutter and pelvis, without gallstones and bile duct dilation. The abdominal tomography also showed the same findings. The initial clinical diagnosis was sepsis due to acalculous cholecystitis and ceftriaxone, metronidazol, platelets transfusion, fluids, antipyretic and analgesic medications were prescribed. An urgent exploratory laparotomy was performed, which showed an edematous gallbladder without signs of acute cholecystitis, and serohematic ascites without any other abnormalities. The patient was transferred to the intensive care unit, and evolved, in the postoperative period, with refractory shock, pronounced lactic acidosis, and multiple organ dysfunction. He was under mechanical ventilation, received vasoactive drugs and other standard intensive care measures. However, the patient died within 48 h of hospitalization. The autopsy was requested by the intensive medical care staff. The laboratory investigation ruled out yellow fever, leptospirosis, and rickettsia infection (Serology, RT-qPCR, and immunohistochemistry in liver tissue). All blood, ascites, and urine cultures were negatives.

### Autopsy findings

**Fig. 1:**
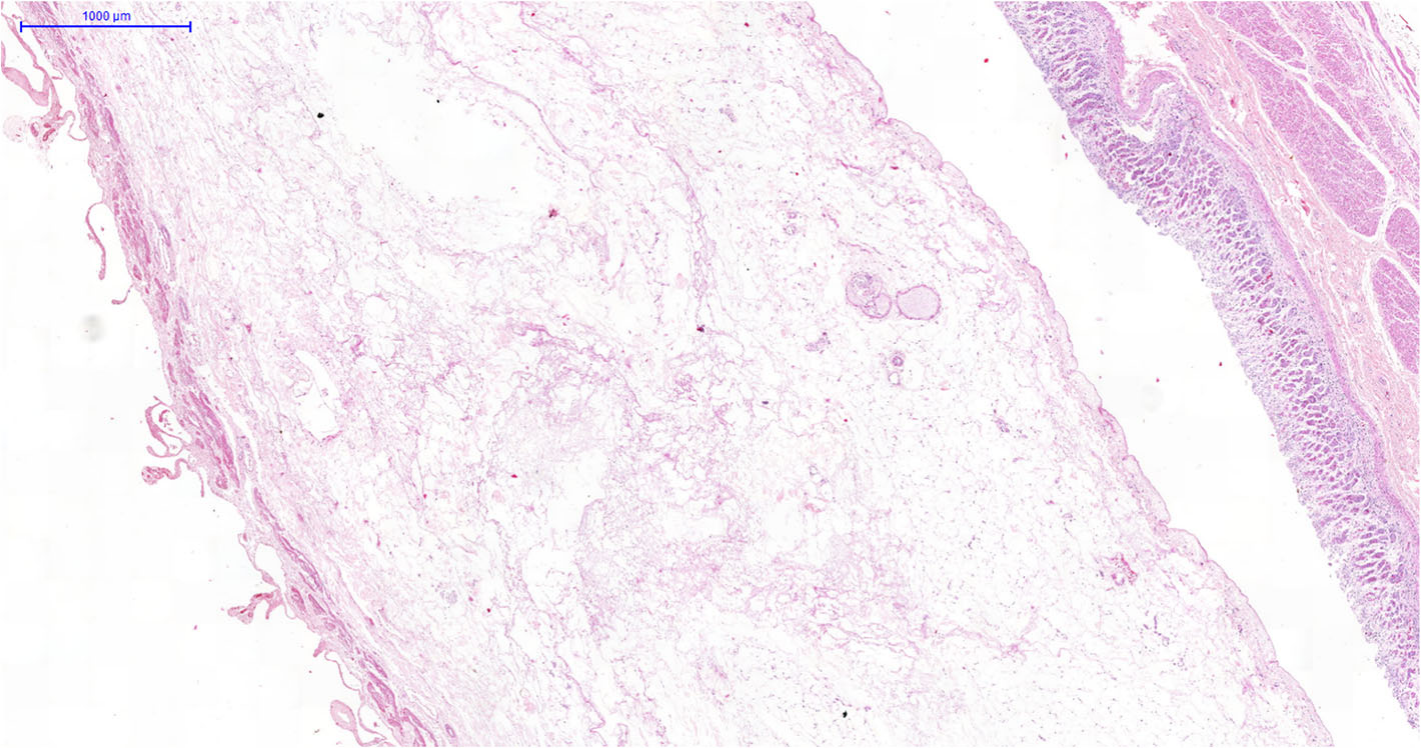
Intense interstitial edema in the gallbladder wall. HE 150x

**Fig. 2:**
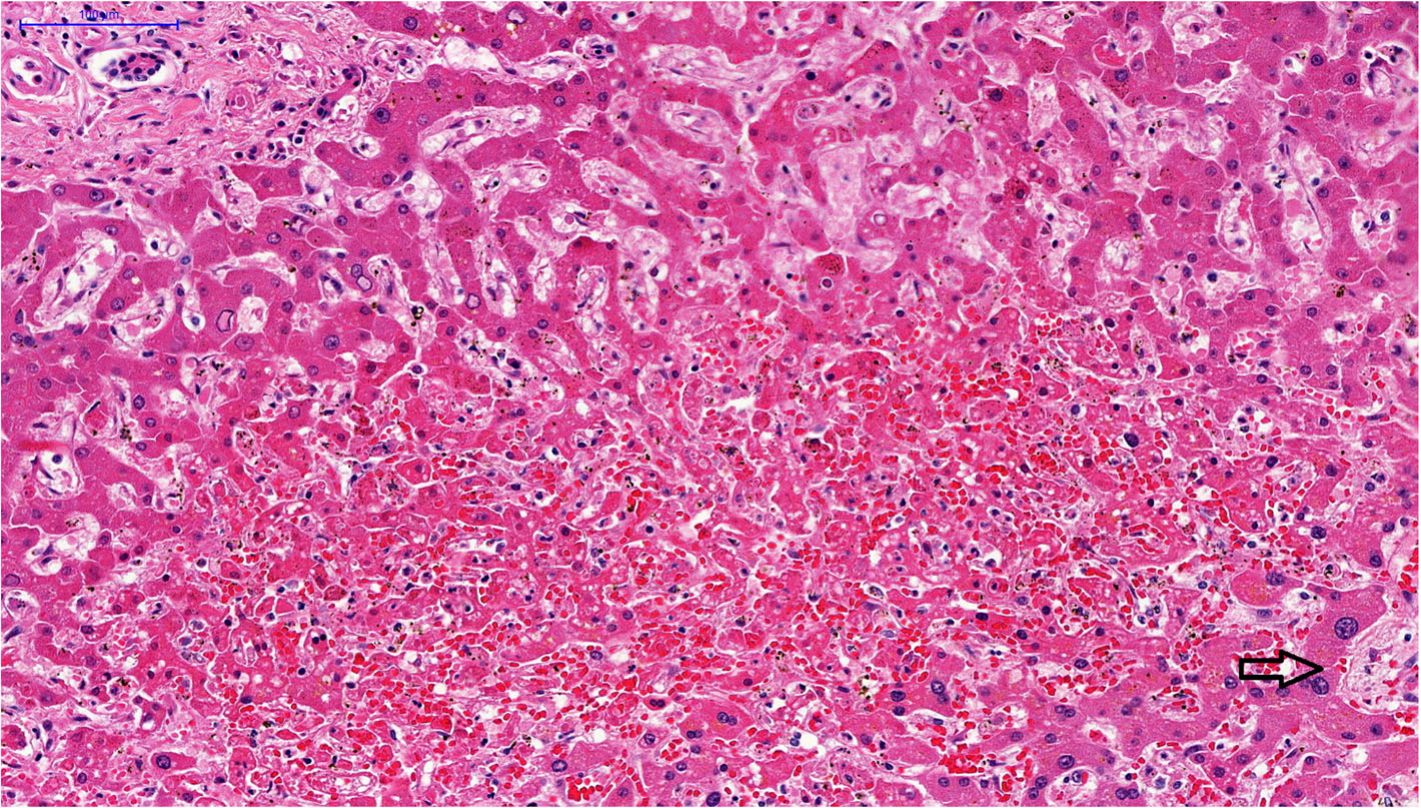
Micrograph of the liver in a fulminant case of dengue fever: midzonal hepatitis, with apoptotic hepatocytes and sinusoidal congestion associated with a scarce inflammatory reaction. The portal area on the left top; arrow indicates centrilobular vein. HE 200x

The main autopsy findings were: bilateral pleural effusion (with serohematic aspect, 1 l each side), ascites, bowel and gallbladder edema (Fig. 1), pulmonary edema and hemorrhage, and concentric left ventricular hypertrophy and atherosclerosis. The esophagus, stomach, and duodenum had hemorrhagic content, with diffuse mucosal bleeding. The liver weighed 906 g (RR = 1650 g) congested and diffusely steatotic. Tissue samples of 1 cm^3^ were collected from the main vital organs for molecular analysis. On microscopy, the liver presented midzonal hepatitis, with apoptotic and steatotic hepatocytes, Kupffer cell hyperplasia, sinus congestion, and hemorrhage, and mild inflammatory reaction, mainly composed of lymphocyte infiltrate (Fig. 2). Other findings were: foci of myocarditis, acute tubular injury, ischemic/reperfusion pancreatitis, spleen with lymphoid hypoplasia, splenitis, and extramedullary hematopoiesis.

### Laboratory and phylogenetic investigation

**Fig. 3:**
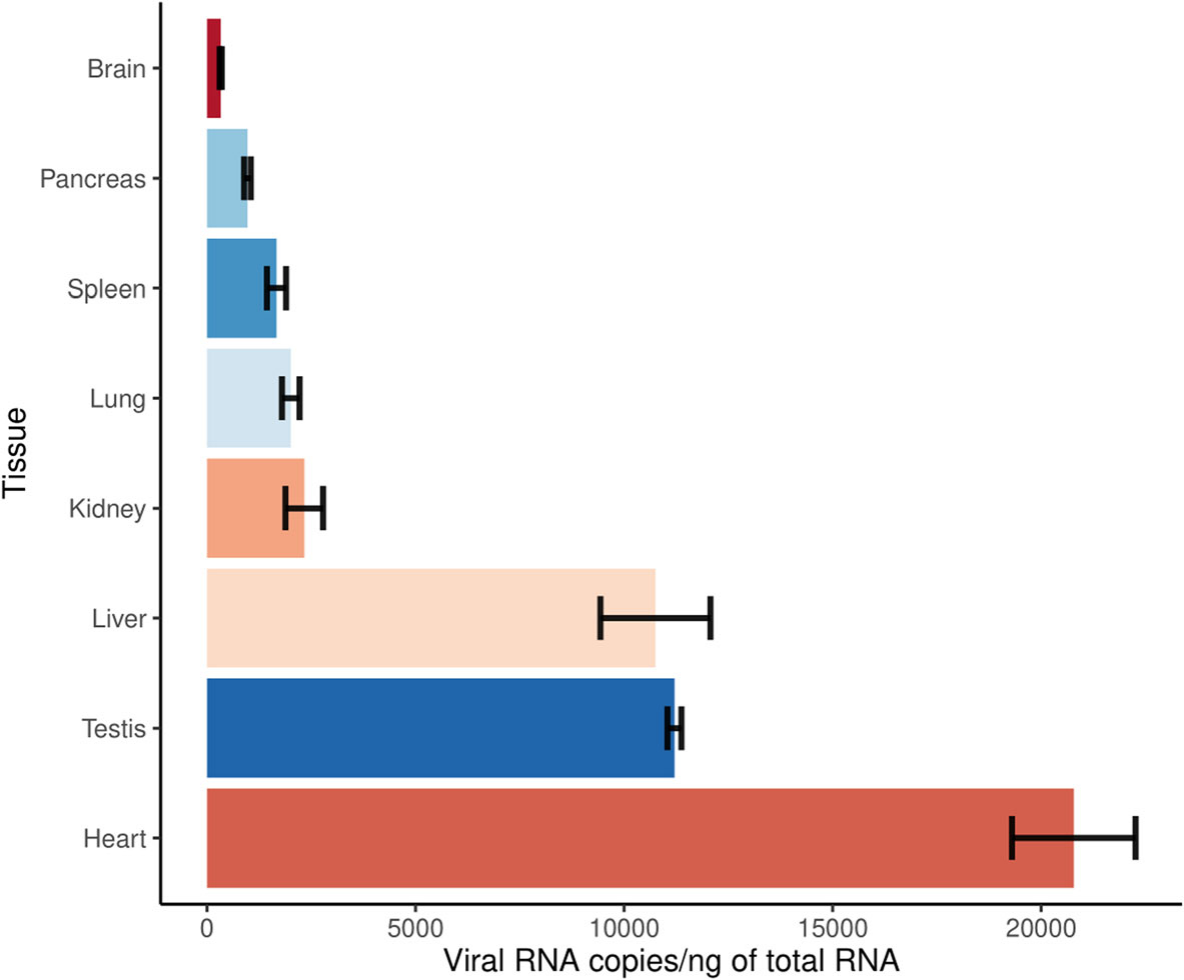
Viral RNA concentration according to each of the 8 tissues analyzed for the patient. The different colors represent the different tissues analyzed, ordered by concentration values. The viral RNA quantification was done in triplicate, with the bar graph representing the average, and the intervals surrounding the mean represent the standard deviation. It is noticeable the high levels of viral RNA in the heart, testis, and liver

**Fig. 4:**
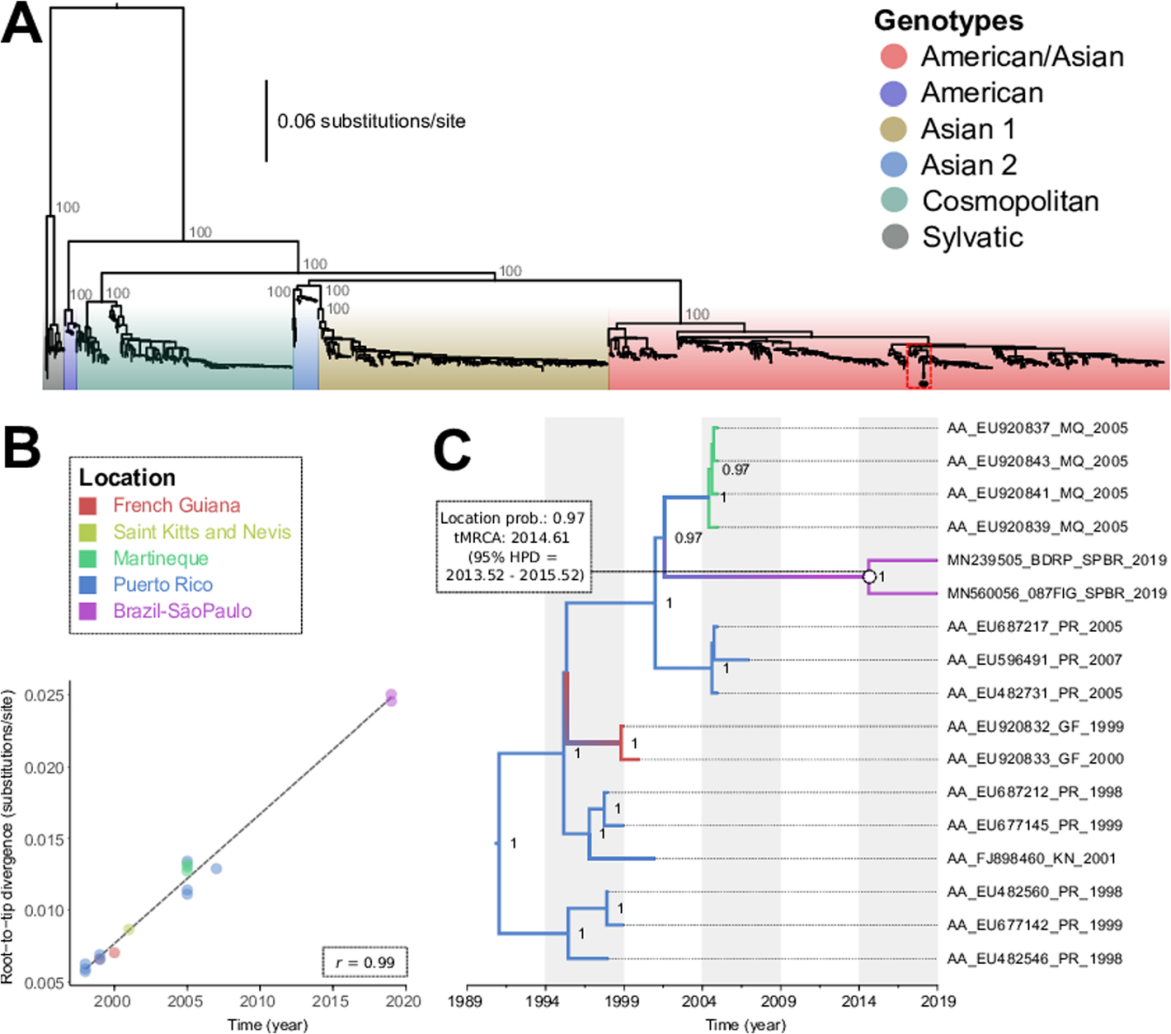
**a** Maximum likelihood phylogenetic trees for DENV-2 based on full-length genome sequences (*n* = 879). The tree is midpoint-rooted and the values near the principal nodes represent statistical support values using the ‘ultrafast’ bootstrap approximation (UFboot) from IQ-TREE. Distinct colors represent different genotypes. The sequences used in phylogeography are marked with the dotted red square in the American/Asian genotype. The two Brazilian sequences isolated in 2019 are highlighted with the black circle. **b** A regression of root-to-tip genetic distance against the time of sampling and showing a positive relationship (*r* = 0.99) indicative of a high rate of evolutionary change over the sampling period. **c** Time-stamped, MCC tree of the DENV-2 American/Asian genotype associated with the Brazilian sequences isolated during 2019 (*n* = 17). The distinct colors represent samples from different locations. The values near the nodes represent posterior probability support

Molecular and serological results point to the patient as positively infected by DENV-2. All collected tissues were positive by RT-qPCR, indicating the presence of viral RNA in the tissues collected. The heart, testis, and liver had the highest amount of viral RNA (Fig. 3). The liver tissue was submitted to viral RNA sequencing, and the libraries were assembled and sequenced on the Illumina platform. The complete genome assembled with high coverage was from a DENV-2 serotype belonging to the American/Asian genotype, as indicated by a maximum likelihood tree (Fig. 4a). The sequence we characterized is closely related to another one also from Brazil and others previously isolated in Puerto Rico (2005–2007), Martinique (2005), and Guadeloupe (2006), available from GenBank. The two Brazilian sequences belong to a group not previously documented in Brazil, and possibly, constitute a new viral introduction from Puerto Rico that took place around 2014.61 (95% HPD = 2013.52–2015.52) (Fig. 4b-c).

## Discussion and conclusion

Usually, clinical manifestations associated with Dengue virus-induced infection vary from asymptomatic, unspecific acute febrile illness to severe dengue fever, which may progress to a fatal outcome [20–22]. By early August, 15,514 autochthonous cases were confirmed in São Paulo city, with 3 deaths and a 0.019 mortality rate [23]. In this regard, we describe a fatal autochthonous case, that occurred in the city of São Paulo, during the 2019 seasonal period caused by a new DENV-2 introduction, originating, most likely, from the Caribbean islands such as Puerto Rico, Martinique, and Guadeloupe. This case was noteworthy since it presented high viral RNA levels in several organs with significant signs of severe damage.

The patient came to the Clinical Hospital of the Faculty of Medicine of the University of São Paulo (HC-FMUSP) during the critical phase of the infection, with warning signs such as serosal effusions, severe abdominal pain, and vomiting, due to plasma leakage, which usually occurs on the 4–6 day of illness [24]. Gallbladder edema and acalculous cholecystitis may be a major manifestation of severe dengue, mimicking an acute abdomen, masking the diagnosis of the disease. Ultrasound can detect acute acalculous cholecystitis and gallbladder edema, which occur in about 5–8% of dengue cases [20, 25], with a higher incidence in children [26]. Thick gallbladder wall has a positive association with Dengue Hemorrhagic Fever in a recent retrospective study conducted in Malaysia [20]. In general, the acalculous cholecystitis is accompanied by fever, Murphy’s sign, and ascites [26]. Without early fluid replacement, patients can evolve to an unfavorable outcome, with shock, as in the present case. The pathological features vary from intense edema of the gallbladder wall, as in our case, to mononuclear cells infiltrate, lymphoid follicle formation, and interstitial hemorrhages [21].

Viral RNA was detected on all collected tissues. This is in line with the previous detection of viral RNA in tissues, such as the kidney, heart, lung, and brain [27]. The concentrations of viral RNA found revealed the systemic spread of the virus to different organs associated with tissue damage. These observations were in line with previously reported lethal cases [28, 29].

Since its first report in Brazil [18], the American/Asian genotype vanished and was reintroduced a few times, always moving from Central America and the Caribbean to Brazil, following a pattern that was well documented for other Dengue serotypes [9, 10, 16]. As we characterize only 2 isolated sequences in the state of São Paulo, the date of introduction of the virus in Brazil we presented may be inaccurate. New sequences from other states, may help clarify this issue. Other studies indicated the circulation of this same virus in Rio de Janeiro and the west region of the state of São Paulo between 2018 and 2019 associated with the current outbreak [30, 31]. It has been argued that the viral genetic diversity in Puerto Rico tends to be maintained in situ, being driven by genetic drift with clade extinction and replacement events over time, with exchanges taking place with South and Central America and other Caribbean regions [32]. The intense movement of emerging viruses is mainly associated with the movement of asymptomatic infected humans and vectors, which is facilitated by rapid transport, such as air transportation [33].

Our results combined with other studies [30, 31] suggested a new DENV-2 introduction in Brazil, that may be associated with the phenomenon known as clade replacement that was demonstrated for other DENV lineages in different locations [16, 34–36]. Other than stochasticity, this could be explained by different factors, such as genetic differences in viral replication in vertebrate and/or invertebrate host [11], or possibly by the fact that herd immunity reduction against DENV-2, since it has not caused detectable outbreaks in Brazil during the last decade [9, 10, 37]. This fatal case associated with a new DENV-2 introduction in Brazil represents a small fraction when compared to classical dengue cases, and the systemic infection in the patient needs to be interpreted with caution, as it is a single-case study, and the patient has previous physiological disorders.

## Supplementary Information

**Additional file 1.**

## Data Availability

All the datasets analyzed during the current study are available from the corresponding author on reasonable request. The new sequence here characterized was deposited in GenBank under the accession number MN560056.

## Abbreviations

DENV: Dengue virus
RNA: Ribonucleic acid
Hb: Hemoglobin
RR: Reference Range
HT: Hematocrit
RT-qPCR: Reverse transcription-polymerase chain reaction quantitative real-time
HPD: Highest posterior density
HC-FMUSP: Clinical Hospital of the Faculty of Medicine of the University of São Paulo
