# Supplementary material for: Systemic dengue infection associated with a new dengue virus type 2 introduction in Brazil – a case report
Source: BMC Infect Dis. 2021 Apr 1;21:311. doi: 10.1186/s12879-021-05959-2 (PMC8015031; doi:10.1186/s12879-021-05959-2)
Supplement: Supplementary file 1 — Additional file 1. [file 12879_2021_5959_MOESM1_ESM.docx]

Supplementary information for:

**Systemic Dengue infection associated with a new Dengue virus type 2 introduction in Brazil – a case report**

Marielton dos Passos Cunha ^#^, Amaro Nunes Duarte-Neto, Shahab Zaki Pour, Ludhmila Abrahão Hajjar, Fernando Pereira Frassetto, Marisa Dolhnikoff, Paulo Hilario do Nascimento Saldiva, Paolo Marinho de Andrade Zanotto ^#^

# **Corresponding author**: marieltondospassos@gmail.com (MPC); pzanotto@usp.br (PMAZ).

**Contents:**

- Material and methods
- Ethical statement
- Dengue – case definition
- Autopsy protocol and tissue processing
- Molecular characterization
- Sequencing and viral genome assembly
- Phylogenetic analysis
- Discrete phylogeographic inference
- Data availability

**Material and methods**

**Ethical statement.** This descriptive study investigates deaths by arbovirus infection from December/2017 to June/2019, conducted by the Pathology Department of the Faculty of Medicine of the University of São Paulo (FMUSP) and by the Laboratory of Molecular Evolution and Bioinformatics (ICB-USP). The research protocol was approved by the Research Ethics Committee of the Clinical Hospital (HC-FMUSP) (CAPPesq #426.643, CAAE protocol number: 18781813.2.0000.0068). Epidemiological, clinical (including demographic data, preexisting medical conditions, clinical signs and symptoms and in-hospital follow-up until death) and laboratory data were collected according to the medical charts. All the information obtained from the patient was anonymized for this report.

**Dengue – case definition.** The case definition of DENV was established by the Brazilian Ministry of Health and the Health Department of São Paulo State and includes: (*i*) suspected cases as patients with fever for a maximum of 7 days, accompanied by at least two of the following symptoms: headache, retro-orbital pain, myalgia, arthralgia, prostration, rash with exposure to an area with dengue transmission or with the presence of *Aedes aegypti* in the last two weeks; (*ii*) confirmed are cases with positive results in diagnostic tests such as real-time–PCR, DENV NS1 antigen detection, IgM antibody detection, cell culture isolation of DENV using the serum, blood, other body fluids or tissue samples when appropriate, as well as histopathology associated with immunohistochemistry technique. The case received the definitive laboratory diagnosis of DENV infection by the Adolfo Lutz Institute (IAL), the State Reference Laboratory, with independent confirmation at the Laboratory of Molecular Evolution and Bioinformatics (LEMB) at the Microbiology Department of the Biomedical Institute of the University of São Paulo (USP). Other acute endemic and seasonal infections were excluded.

**Autopsy protocol and tissue processing.** The autopsy was performed after consent of the family members and following the Letulle technique, where all the organs were removed *en masse* (one block), requiring dissection organ by organ to exam them individually. Samples, measuring 1 cm^3^, were collected from the: (*i*) heart, (*ii*) lung, (*iii*) brain, (*iv*) kidney, (*v*) spleen, (*vi*) pancreas, (*vii*) liver, and (*viii*) testis, and were stored in -80°C for the RT-qPCR procedures.

**Molecular characterization.** Nucleic acid extraction from all collected tissues was performed using the TRIzol® reagent (Life Technologies, Carlsbad, CA, USA) and carried out according to the manufacturer’s instructions. Molecular detection of DENV was performed with the use of the AgPath-ID One-Step RT-PCR Reagents (Ambion, Austin, TX, USA) with specific primers/probe previously described [1]. To quantify the viral RNA concentration in each tissue, we used a double-stranded DNA fragment (gBlocks) (IDT, Coralville, IA) containing the amplification region, which from several known molecules, was diluted and used to estimate the viral RNA concentration by linear regression. All the RT-qPCR reactions consisted of a step of reverse transcription at 45°C for 10 min, enzyme activation at 95°C for 10 min, and 40 cycles at 95°C for 15 s and 60°C for 45 s for hybridization and extension using the ABI7500 equipment (Thermo Fisher Scientific, Waltham, MA, USA).

**Sequencing and viral genome assembly.** Based on the RNA viral concentration, total RNA were extracted from the liver tissue using the TRIzol® reagent (Life Technologies, Carlsbad, CA, USA). Subsequently, the RNA was purified with DNase I and concentrated using the RNA Clean and Concentrator ^TM-5^ kit (Zymo Research, Irvine, CA, USA) according to the manufacturer’s instructions. The paired-end RNA libraries were constructed and validated using the TruSeq Stranded Total RNA HT sample prep kit (Illumina, San Diego, CA, USA). Sequencing was done at the Core Facility for Scientific Research – University of São Paulo (CEFAP-USP/GENIAL) using the Illumina MiSeq platform. Short unpaired reads and low-quality bases and reads were removed using Trimmomatic version 0.36 (LEADING:20 TRAILING:20 SLIDINGWINDOW:4:25 MINLEN:36) [2]. The genome assembly was performed using the *de novo* approach with the SPAdes v3.13.1 program [3] using default parameters.

**Phylogenetic analysis.** Phylogenetic inference was performed with a previously curated dataset [4] updated with DENV-2 Brazilian complete genome sequences isolated in 2018-2019 available from GenBank (until October 2019). Our new DENV-2 genome was aligned to other reference sequences using Clustal Omega (dataset-1) [5]. A phylogenetic tree was reconstructed based on full-length, curated DENV-2 coding sequences using the Maximum Likelihood (ML) method implemented in IQ-TREE 1.5.5 [6] with automatic model selection by ModelFinder and using the Bayesian Information Criterion (BIC) [7]. The robustness of the groupings observed was assessed using an ultrafast bootstrap approximation (UFboot) during 1,000 replicates. The ML tree was visualized and plotted using FigTree v.1.4.3 [8]. Taxon labels for sequences used in this work had the format: genotype/accession number/local of isolation/date of isolation.

**Discrete phylogeographic inference**

Based on the topology of the phylogenetic tree, we chose a group of basal and previous isolated sequences in Central America to reconstruct the viral movement and trace the origin of the Brazilian sequences (dataset-2). We explored the temporal signal (i.e., molecular clock structure) and quality of our data set using TempEst v.1.5.3 [9]. The spatiotemporal spread was reconstructed under a Bayesian framework implemented in BEAST v.1.10.4 [10]. For comparisons, we tested the molecular clock under (*i*) a strict molecular clock model that assumes that every branch in a phylogenetic tree evolves according to the same evolutionary rate; and (*ii*) an uncorrelated relaxed molecular clock that allows for each branch of a phylogenetic tree to have its evolutionary rate [11]. In our phylogeographic dataset, the marginal posterior distribution of the coefficient of variation of the uncorrelated relaxed molecular (log-normal distribution) (UCLN) model did not exclude zero. Therefore, a strict molecular model was used during the subsequent analyses. We tested a strict clock in combination with non-parametric population growth models: (*i*) the standard Bayesian skyline plot (BSP; 10 groups) [12], (*ii*) the Bayesian skyride plot [13], and (*iii*) the Bayesian skygrid model [12] (Supplementary Table 1). Phylogeography patterns and parameters were estimated running a Markov Chain Monte Carlo (MCMC) for 50 million states, and sampling every 50.000 states with 10 % burnin. Convergence and the effective sample size (ESS) >200 were examined with Tracer v.1.7.1 [14]. The maximum clade credibility (MCC) tree was also visualized and edited with FigTree v.1.4.3 [8]. To calculate the log marginal likelihood for molecular clock and demographic model selection, we used the path sampling (PS) and the stepping-stone (SS) sampling approaches by running 100 path steps of 1 million iterations each.

**Data availability.** The new sequence here characterized was deposited in GenBank under the accession number MN560056.

**References**

1. Wagner D, De With K, Huzly D, Hufert F, Weidmann M, Breisinger S, et al. Nosocomial acquisition of dengue. Emerg Infect Dis. 2004;10:1872–3.

2. Bolger AM, Lohse M, Usadel B. Trimmomatic: A flexible trimmer for Illumina sequence data. Bioinformatics. 2014;30:2114–20.

3. Bankevich A, Nurk S, Antipov D, Gurevich AA, Dvorkin M, Kulikov AS, et al. SPAdes: A New Genome Assembly Algorithm and Its Applications to Single-Cell Sequencing. J Comput Biol. 2012;19:455–77.

4. Cunha M dos P, Ortiz-Baez AS, Freire CC de M, Zanotto PM de A. Codon adaptation biases among sylvatic and urban genotypes of Dengue virus type 2. Infect Genet Evol. 2018;64 May:207–11. doi:10.1016/j.meegid.2018.05.017.

5. Larkin MA, Blackshields G, Brown NP, Chenna R, Mcgettigan PA, McWilliam H, et al. Clustal W and Clustal X version 2.0. Bioinformatics. 2007;23:2947–8.

6. Nguyen L-T, Schmidt HA, Haeseler A von, Minh BQ. IQ-TREE: A Fast and Effective Stochastic Algorithm for Estimating Maximum-Likelihood Phylogenies. Mol Biol Evol. 2015;32:268–74. doi:10.1093/molbev/msu300.

7. Kalyaanamoorthy S, Minh BQ, Wong TKF, Von Haeseler A, Jermiin LS. ModelFinder: Fast model selection for accurate phylogenetic estimates. Nat Methods. 2017;14:587–9. doi:10.1038/nmeth.4285.

8. Rambaut A, Drummond A. FigTree. 2009.

9. Rambaut A, Lam TT, Max Carvalho L, Pybus OG. Exploring the temporal structure of heterochronous sequences using TempEst (formerly Path-O-Gen). Virus Evol. 2016;2:vew007. doi:10.1093/ve/vew007.

10. Suchard MA, Lemey P, Baele G, Ayres DL, Drummond AJ, Rambaut A. Bayesian phylogenetic and phylodynamic data integration using BEAST 1.10. Virus Evol. 2018;4:1–5.

11. Drummond AJ, Ho SYW, Phillips MJ, Rambaut A. Relaxed phylogenetics and dating with confidence. PLoS Biol. 2006;4:699–710.

12. Gill MS, Lemey P, Faria NR, Rambaut A, Shapiro B, Suchard MA. Improving bayesian population dynamics inference: A coalescent-based model for multiple loci. Mol Biol Evol. 2013;30:713–24.

13. Minin VN, Bloomquist EW, Suchard MA. Smooth skyride through a rough skyline: Bayesian coalescent-based inference of population dynamics. Mol Biol Evol. 2008;25:1459–71.

14. Rambaut A, Drummond AJ. Tracer v1.4. Available from http://beast.bio.ed.ac.uk/Tracer. Available from http://beast.bio.ed.ac.uk/Tracer. 2007.

**Supplementary Table 1**. Model comparison of the strict molecular clock and demographic growth models through path sampling (PS) and stepping stone (SS) methods. Bold numbers indicate the best fitting model.

|  | **Strict molecular clock** | |
| --- | --- | --- |
| **Demographic growth model** | **PS** | **SS** |
| **Bayesian skyline plot** | **-17846.23** | **-17846.20** |
| Bayesian skyride plot | -17847.48 | -17847.59 |
| Bayesian skygrid model | -17847.37 | -17847.37 |

PS: Path sampling; SS: Stepping stone.
